# African swine fever virus pMGF505-9R enhances RIG-I-like receptor signaling by promoting RING finger protein 125 autoubiquitination

**DOI:** 10.1016/j.jbc.2025.110669

**Published:** 2025-08-30

**Authors:** Jinyu Zhao, Xinyu Zhang, Chunxiao Mou, Kaichuang Shi, Zhenhai Chen

**Affiliations:** 1 College of Veterinary Medicine, Yangzhou University, Yangzhou, Jiangsu Province, China; 2 Guangxi Center for Animal Disease Control and Prevention, Nanning, Guangxi Province, China; 3 Joint International Research Laboratory of Agriculture and Agri-Product Safety, the Ministry of Education of China, Yangzhou University, Yangzhou, Jiangsu Province, China; 4 Jiangsu Co-Innovation Center for Prevention and Control of Important Animal Infectious Diseases and Zoonoses, Yangzhou University, Yangzhou, Jiangsu Province, China

**Keywords:** ASFV, pMGF505-9R, RLR, RNF125, autoubiquitination

## Abstract

African swine fever (ASF) is a highly infectious disease that poses a significant threat to the global pig industry. Recent studies have demonstrated that the African swine fever virus (ASFV) infection can cause severe inflammatory responses and promote the production of cytokines, but it is still unclear whether the viral proteins play a role in this process. Therefore, we conducted a genome-wide screening by dual luciferase activity assay. The results showed that six viral proteins of ASFV have a stimulating effect on the promoter activity of interferon beta (IFN-β). Among them, the nonstructural protein pMGF505-9R significantly increased the activity of IFN-β promoter and promoted the expression of IFN-β. In addition, pMGF505-9R can also increase the promoter activity of NF-κB and promoted the expression of interleukin 1 beta (IL-1β). Further research found that pMGF505-9R could regulate the retinoic acid–inducible gene I–like receptor signaling pathways by influencing the protein level of RING finger protein 125 (RNF125), ultimately affecting the expression of IFN-β and IL-1β. Mechanistically, pMGF505-9R interacted with the host E3 ubiquitin ligase RNF125. This interaction promoted the autoubiquitination and subsequent degradation of RNF125, which in turn reduced the K48 ubiquitination of retinoic acid–inducible gene I, melanoma differentiation–associated protein 5, and mitochondrial antiviral signaling, which ultimately promoted the production of IFN-β and IL-1β. The results provide novel insights into the immune regulatory mechanisms of ASFV, which would greatly improve our understanding of virus pathogenesis.

African swine fever virus (ASFV) is a large nucleocytoplasmic DNA virus that has a genome of approximately 170 to 190 kb and encodes over 160 proteins. Currently, the functions of more than half of these viral proteins remain unidentified. The ASFV particle exhibits a regular icosahedral symmetry, with an average diameter of 200 to 300 nm. The mature virus particle comprises five components: the outer membrane, capsid, inner membrane, nucleocapsid, and viral core (1, 2, 3, 4). ASF was first identified in Kenya during the 1920s and became widespread in Portugal and Spain by the mid-20th century, subsequently spreading to Europe and South America. In the 21st century, ASF has extended its reach to several countries in Eastern Europe and Asia (5). Current research on ASFV vaccine development primarily focuses on live-attenuated vaccine platforms, with significant breakthroughs achieved: Studies have confirmed that targeted deletion of six immunomodulatory genes (MGF360-12L, 13L, 14L and MGF505-1R, 2R, 3R) can completely attenuate the virulence of the Georgia strain (6), whereas another study demonstrated that deletion of the I177L gene effectively reduces pathogenicity in the same strain (7). These findings collectively demonstrate the safety advantages of attenuated viral variants and their potential as promising vaccine candidates. Therefore, identifying immune regulatory genes in ASFV and constructing gene-deleted viral strains provides a robust foundation for clinical vaccine development.

As the first line of defense against pathogen invasion, innate immunity recognizes specific pathogen-associated molecular patterns through pattern recognition receptors, thereby triggering innate immune responses. For DNA viruses, their DNA can be detected by host DNA sensors, such as cyclic GMP–AMP synthase. Conversely, the nucleic acids of RNA viruses are primarily recognized by retinoic acid–inducible gene I (RIG-I) and melanoma differentiation–associated protein 5 (MDA5) (8, 9). However, there are exceptions: for instance, DNA-dependent RNA polymerase III transcribes AT-rich DNA templates to produce 5′-triphosphate RNA (5′-ppp-RNA), which is subsequently recognized by RIG-I (10, 11). The AT-rich genome of ASFV makes it possible for RIG-I to recognize its transcribed RNA (12). This mechanism facilitates a more comprehensive antiviral response, ensuring that both DNA and RNA viruses are effectively detected and countered by the host immune system.

Existing research shows that some viral proteins also have immune-stimulating effects, such as the interaction between the dengue virus membrane protein (M) and the host's NLRP3 (NLR family domain containing 3) protein, which promotes the activation of the NLRP3 inflammasome (13), and the severe acute respiratory syndrome coronavirus 2 S protein and NSP14 promote the activation of NF-κB (14, 15). In the case of ASFV, although numerous ASFV proteins with immune evasion functions have been identified, clinical data indicate that the ASFV infection can cause severe inflammatory responses (16, 17). The findings suggest that ASFV may not only negatively regulate the host's innate immune response but also exert a positive regulatory effect.

In the present study, we found that pMGF505-9R could enhance the RIG-I-like receptor (RLR) signaling pathway by antagonizing the negative regulatory factor RING finger protein 125 (RNF125). This study provides new insights into the immune regulatory mechanisms of ASFV.

## Results

### Interferon and inflammatory responses are activated in porcine alveolar macrophages exposed to ASFV

**Figure 1:**
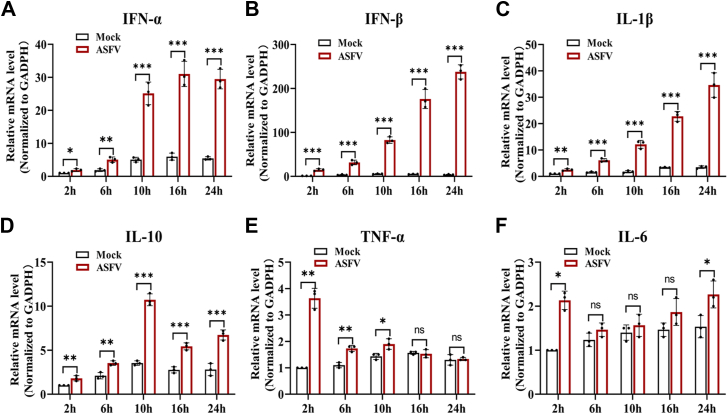
**IFN and inflammatory responses are activated in porcine alveolar macrophages (PAMs) exposed to African swine fever virus.***A*–*F*, PAMs were infected with ASFV at an MOI of 1, and RNA was extracted from the cells at 0, 2, 6, 10, 16, and 24 hpi. The mRNA levels of IFN-α, IFN-β, IL-1β, IL-10, TNF-α, and IL-6 were detected using quantitative PCR, and relative mRNA levels were normalized to GAPDH. ASFV, African swine fever virus; IFN, interferon; IL, interleukin; MOI, mechanism of action; TNF, tumor necrosis factor.

ASFV infection causes a variety of inflammatory conditions, including splenomegaly, pulmonary edema, and hemorrhage. To investigate the impact of ASFV infection on type I interferon (IFN) and inflammatory responses, porcine alveolar macrophages (PAMs) were infected with the ASFV strain CN/JS-1/2024. Subsequently, mRNA levels of IFN-α, IFN-β, interleukin 1 beta (IL-1β), IL-10, tumor necrosis factor-α, and IL-6 in cells were measured at 2, 6, 10, 16, and 24 hpi. The results showed that during ASFV infection, the expression of IFN-α, IFN-β, and IL-1β gradually increased in a time-dependent manner (Fig. 1, *A*–*C*). In addition, the expression of IL-10 reached its peak at the 10 hpi (Fig. 1*D*), whereas the expression levels of tumor necrosis factor-α and IL-6 reached their maximums at the 2 hpi (Fig. 1, *E* and *F*). These findings suggested that ASFV infection could activate type I IFN and inflammatory responses.

### pMGF505-9R promoted the production of IFN-β and IL-1β

**Figure 2:**
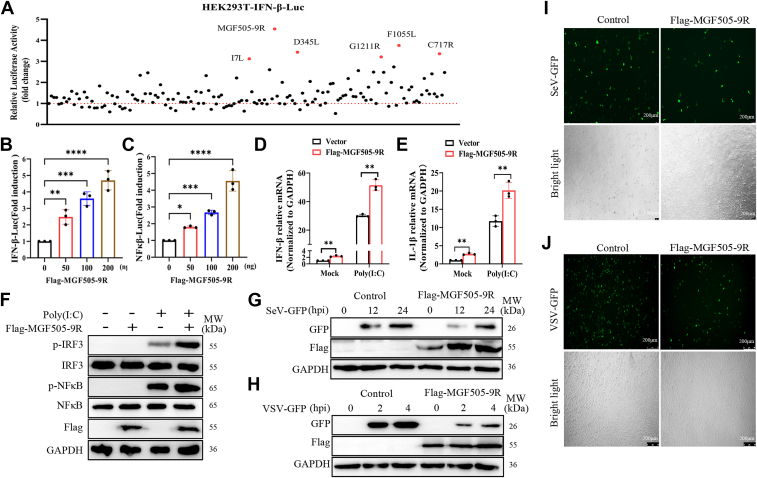
**pMGF505-9R promoted the production of IFN-β and IL-1β**. *A*, HEK-293T cells were transfected with a plasmid expressing one of the ASFV-encoded proteins, along with 100 ng of a reporter plasmid and 5 ng of the pRL-TK plasmid. At 36 h post-transfection, the cells were collected, and the activation of the IFN-β promoter was determined using the Dual-Luciferase assay kit. *B* and *C*, HEK-293T cells were transfected with 0.01 mg/well of pRL-TK plasmid, along with 0.1 mg/well of either the IFN-β-Luc, NF-κB-Luc, or ISRE-Luc plasmids, as well as increasing doses (0, 50, 100, and 200 ng) of the FLAG-MGF505-9R expression plasmid. At 36 h post-transfection, the activation of the respective promoters was determined using the Dual-Luciferase assay kit. *D* and *E*, ST cells were transfected with poly(I:C) and FLAG-MGF505-9R expression plasmid or an empty vector. At 24 hpt, the mRNA levels of IFN-β and IL-1β were analyzed by quantitative PCR. *F*, ST cells were transfected with poly(I:C) and FLAG-MGF505-9R expression plasmid or an empty vector for 24 h, and the protein levels and phosphorylation levels of IRF3 and NF-κB were analyzed by Western blotting. *G* and *H*, ST cells and 293A cells were transfected with the MGF505-9R expression plasmid for 12 h and then infected with SeV-GFP or VSV-GFP at an MOI of 0.1 at the indicated times. The cell lysates were analyzed by immunoblotting using antibodies against FLAG, GFP, or GAPDH. *I* and *J*, ST cells and 293A cells were transfected with the MGF505-9R expression plasmid for 24 h and then infected with SeV-GFP at an MOI of 0.1 for 12 h or VSV-GFP at an MOI of 0.1 for 4 h, then the fluorescence images were captured using fluorescence microscopy. The scale bar represents 200 μm (SeV) and 100 μm (VSV). ASFV, African swine fever virus; HEK-293T, human embryonic kidney 293T; IFN, interferon; IL, interleukin; IRF3, interferon regulatory factor 3; MOI, multiplicity of infection; SeV, Sendai virus; VSV, vesicular stomatitis virus.

Viruses modulate the host's innate immune response to facilitate their replication. Recent research has shown that some viral proteins can positively regulate the NF-κB pathway (18, 19, 20). ASFV encodes more than 160 viral proteins, but whether these proteins have the ability to upregulate innate immune signaling pathways remains unclear. Therefore, we conducted whole-genome sequencing of the ASFV strain CN/JS-1/2024 and constructed a eukaryotic expression library for the proteins encoded by this strain. Then, we screened all 160 ASFV proteins using a dual luciferase activity assay to identify those with immunostimulatory effects. The results showed that six viral proteins significantly upregulated the promoter activity of IFN-β, with pMGF505-9R having the most significant effect (Fig. 2*A*). Further investigations revealed that pMGF505-9R not only increased the activity of the IFN-β promoter but also enhanced the activity of NF-κB promoters in a dose-dependent manner (Fig. 2, *B* and *C*). To determine whether pMGF505-9R influences the expression of IFN-β and IL-1β, we measured the mRNA levels of these genes in cells transfected with MGF505-9R expression plasmids and poly(I:C). The results showed that pMGF505-9R could upregulate poly(I:C)-induced mRNA expression of both IFN-β and IL-1β (Fig. 2, *D* and *E*), and Western blot analysis revealed that pMGF505-9R significantly upregulated the phosphorylation levels of NF-κB and interferon regulatory factor 3 (IRF3) (Fig. 2*F*). Given that pMGF505-9R positively regulated the RLR signaling pathway, we then examined the effect of pMGF505-9R on the replication of IFN-sensitive viruses, Sendai virus (SeV) and vesicular stomatitis virus (VSV). As expected, pMGF505-9R significantly inhibited the replication of SeV and VSV (Fig. 2, *H*–*K*). In conclusion, these results indicated that pMGF505-9R promoted the activation of the RLR signaling pathway, thereby positively regulating cellular antiviral responses.

### pMGF505-9R promoted the activation of RIG-I-like receptor signaling pathway

**Figure 3:**
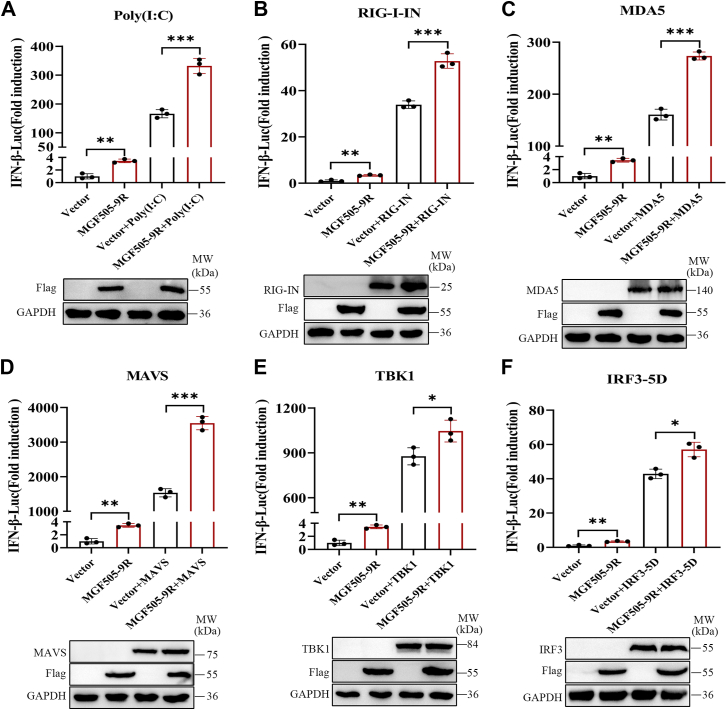
**pMGF505-9R promoted the activation of RIG-I-like receptor signaling pathway.***A*–*F*, HEK-293T cells were transfected with 0.1 mg/well of the IFN-β-Luc expression plasmid, 0.01 mg/well of the pRL-TK plasmid, and either 0.1 mg/well of the vector or the FLAG-505-9R expression plasmid, along with poly(I:C), HA-tagged RIG-I, MDA5, MAVS, TBK1, or IRF3-5D expression plasmids. The activation of the IFN-β promoter was tested using the Dual-Luciferase assay at 24 hpt; HEK-293T, human embryonic kidney 293T cell line; IFN, interferon; IRF3, interferon regulatory factor 3; MAVS, mitochondrial antiviral signaling; MDA5, melanoma differentiation–associated protein 5; RIG-I, retinoic acid–inducible gene I; TBK1, TANK-binding kinase 1.

pMGF505-9R is a member of the MGF505 family, whose proteins mainly have immunosuppressive effects. To verify the enhancing effect of pMGF505-9R on the activation of the IFN-β promoter, we examined the effect of pMGF505-9R on the activity of the IFN-β promoter induced by poly(I:C), RIG-I-IN, MDA5, mitochondrial antiviral signaling (MAVS), TANK-binding kinase 1 (TBK1), and the activated mutant of IRF3 (IRF3-5D). The results showed that pMGF505-9R significantly enhanced the promoter activity induced by poly(I:C), RIG-I-IN, MDA5, and MAVS (Fig. 3, *A*–*D*). In addition, pMGF505-9R slightly increased the IFN-β promoter activity induced by TBK1 and IRF3-5D (Fig. 3, *E* and *F*). These results suggested that pMGF505-9R likely acts upstream of TBK1 to positively regulate the promoter activity of IFN-β.

### pMGF505-9R increased the expression of RIG-I, MDA5, and MAVS by reducing their K48-linked ubiquitination

**Figure 4:**
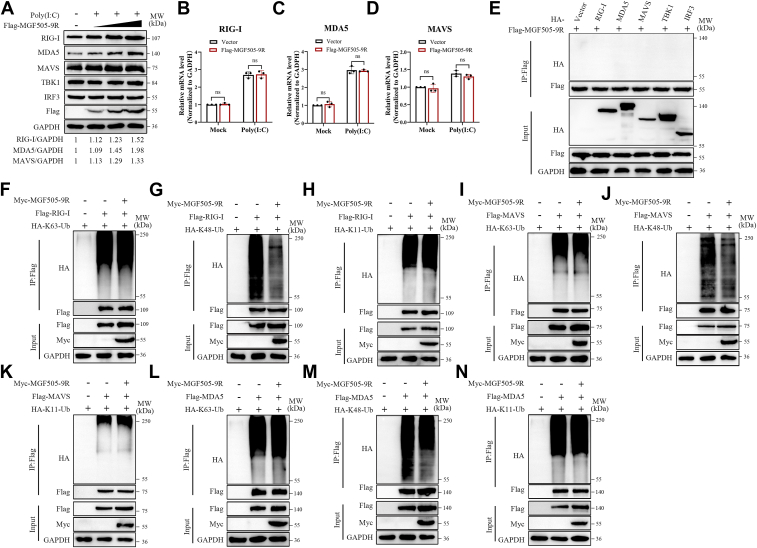
**pMGF505-9R increased the expression of RIG-I, MDA5, and MAVS by reducing their K48-linked ubiquitination.***A*, ST cells were transfected with poly(I:C) and increasing doses of FLAG-MGF505-9R or an empty vector for 24 h, and the protein levels of RIG-I, MDA5, MAVS, TBK1, and IRF3 were analyzed by Western blotting. *B*–*D*, ST cells were transfected with poly(I:C) and FLAG-MGF505-9R or an empty vector for 24 h, and the mRNA levels of RIG-I, MDA5, and MAVS were analyzed by RT–quantitative PCR. *E*, HEK-293T cells were transfected with increasing doses of FLAG-MGF505-9R expression plasmid (0, 0.5, 1, and 2 μg). At 24 hpt, the cells were transfected with poly(I:C) for another 12 h. The levels of MAVS and FLAG-MGF505-9R proteins were analyzed by Western blotting. *D*, HEK-293T cells were transfected with FLAG-MGF505-9R and HA-RIG-I, HA-MDA5, HA-MAVS, HA-TBK1, or HA-IRF3. At 24 hpt, co-IP was performed to detect the interaction between pMGF505-9R and these proteins. *F*–*N*, HEK-293T cells were transfected with plasmids expressing Myc-MGF505-9R, HA-tagged ubiquitin chains (HA-K63-Ub, HA-K48-Ub, or HA-K11-Ub), and either HA-RIG-I, HA-MDA5, or HA-MAVS for 24 h. Co-IP and immunoblotting analyses were performed with the indicated antibodies. Co-IP, coimmunoprecipitation; HEK-293T, human embryonic kidney 293T cell line; IRF3, interferon regulatory factor 3; MAVS, mitochondrial antiviral signaling; MDA5, melanoma differentiation–associated protein; RIG-I, retinoic acid–inducible gene I; TBK1, TANK-binding kinase 1; Ub, ubiquitin.

Based on the aforementioned results, we examined the effect of pMGF505-9R on the protein levels of components in the RLR signaling pathway, including RIG-I, MDA5, MAVS, TBK1, and IRF3. The results showed that pMGF505-9R significantly increased the protein level of RIG-I, MDA5, and MAVS in a dose-dependent manner (Fig. 4*A*). However, there was no change in the mRNA level of RIG-I, MDA5, and MAVS (Fig. 4, *B*–*D*). Furthermore, we investigated the potential interaction between pMGF505-9R and these key components of the RLR signaling pathway, using coimmunoprecipitation (co-IP) assays. The results showed no interaction between pMGF505-9R and any of these proteins (Fig 4*E*). Given the lack of interaction between the proteins and the absence of significant differences in mRNA levels, we hypothesized that post-translational modifications might mediate the observed effects. Prior studies have established that K48-linked ubiquitination, catalyzed by specific E3 ubiquitin ligases, promotes proteasomal degradation of RIG-I, MDA5, and MAVS (5, 21, 22). We therefore examined whether pMGF505-9R modulates K48-linked ubiquitination of these proteins. The results showed that pMGF505-9R could reduce the K48 ubiquitination modification of RIG-I, MDA5, and MAVS but had no effect on their K63 and K11 ubiquitination modifications (Fig. 4, *F*–*N*). Based on the aforementioned results, we can conclude that pMGF505-9R increased the protein level of RIG-I, MDA5, and MAVS by reducing their K48-linked ubiquitination.

### pMGF505-9R impaired the association of RNF125 with RIG-I, MDA5, and MAVS

**Figure 5:**
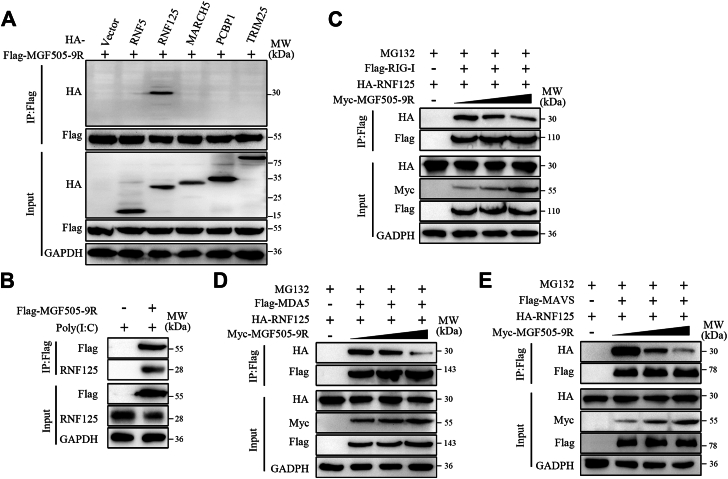
**pMGF505-9R impaire1d the association of RNF125 with RIG-I, MDA5 and MAVS.***A*, HEK-293T cells were cotransfected with FLAG-tagged MGF505-9R and HA-tagged vector, HA-tagged RNF5, HA-tagged RNF125, HA-tagged MARCH5, HA-tagged PCBP1, or HA-tagged TRIM25 for 24 h. Co-IP and immunoblotting were then performed using the indicated antibodies. *B*, ST cells were transfected with poly(I:C) and FLAG-MGF505-9R for 24 h. Co-IP and immunoblotting were then performed using the indicated antibodies. *C*–*E*, HEK-293T cells were cotransfected with increasing doses of Myc-MGF505-9R, HA-RNF125, and FLAG-RIG-I, FLAG-MDA5, or FLAG-MAVS for 24 h, followed by a 4-h treatment with MG132. Co-IP and immunoblotting were then performed using the indicated antibodies. Co-IP, coimmunoprecipitation; HEK-293T, human embryonic kidney 293T cell line; MAVS, mitochondrial antiviral signaling; MDA5, melanoma differentiation–associated protein 5; RIG-I, retinoic acid–inducible gene I; RNF125, RING finger protein 125.

Multiple E3 ubiquitin protein ligases, such as RNF125, TRIM25, and MARCH5, have been reported to mediate the K48 ubiquitination and degradation of RIG-I, MDA5, and MAVS (23, 24, 25). We speculate that pMGF505-9R may regulate the K48 ubiquitination of RIG-I, MDA5, and MAVS by affecting the activity of some E3 ubiquitin ligases. Therefore, we tested whether pMGF505-9R interacts with the E3 ubiquitin ligases that regulate the K48 ubiquitination of these proteins by co-IP assays. The results demonstrated that pMGF505-9R could interact with overexpressed RNF125 (Fig. 5*A*). Moreover, their interaction was confirmed through co-IP assays to detect endogenous RNF125 in cells (Fig. 5*B*). In addition, we further investigated whether MGF505-9R affects the interactions between RNF125 and its substrates, including RIG-I, MDA5, and MAVS. The co-IP assays revealed that pMGF505-9R dose-dependently inhibited the interaction between RNF125 and RIG-I, MDA5, or MAVS (Fig. 5, *C*–*E*). Collectively, the aforementioned results demonstrated that pMGF505-9R interacted with RNF125 and inhibited the binding of RNF125 to its substrates.

### pMGF505-9R promoted the autoubiquitination and degradation of RNF125

**Figure 6:**
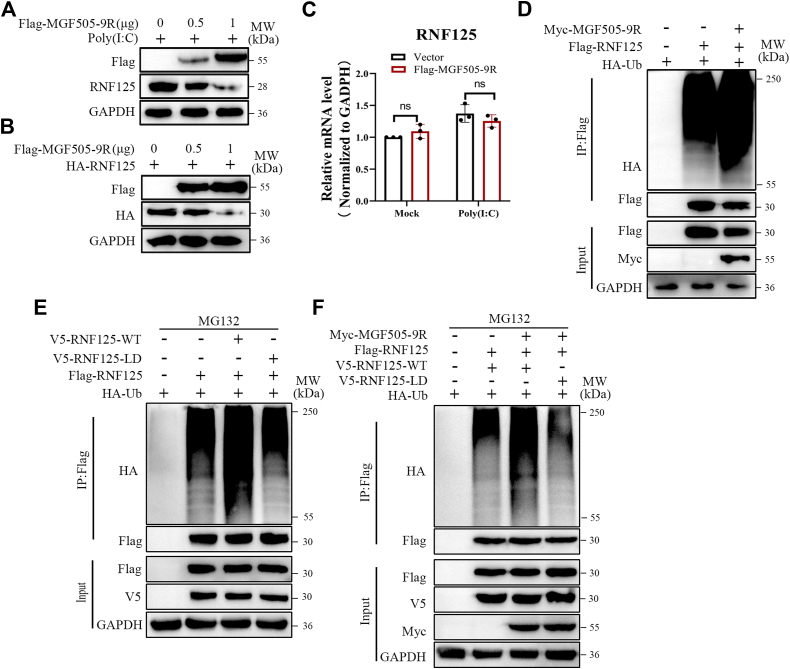
**pMGF505-9R promoted the autoubiquitination and degradation of RNF125.***A*, ST cells were transfected with poly(I:C) and increasing doses of the FLAG-MGF505-9R expression plasmid (0, 0.5, and 1 μg). After 24 hpt, the protein levels of RNF125 and FLAG-MGF505-9R were then analyzed by Western blotting. *B*, HEK-293T cells were transfected with the HA-RNF125 (1 μg) expression plasmid and increasing doses of the FLAG-MGF505-9R expression plasmid (0, 0.5, and 1 μg) for 36 h. The levels of RNF125 and FLAG-MGF505-9R proteins were analyzed by Western blotting. *C*, ST cells were transfected with either the FLAG-MGF505-9R expression plasmid or an empty vector for 24 h, and then the mRNA levels of RNF125 were analyzed by quantitative PCR. *D*, HEK-293T cells were transfected with Myc-MGF505-9R (2 μg), HA-Ub (1 μg), and FLAG-RNF125 (2 μg) for 24 h. Co-IP and immunoblotting assay were performed using the indicated antibodies. *E*, HEK-293T cells were cotransfected with plasmids encoding FLAG-RNF125, HA-Ub, and either V5-RNF125-WT or V5-RNF125-LD (LD: ligase dead) for 24 h, followed by a 4-h treatment with MG132. Subsequently, co-IP and immunoblotting were performed using the indicated antibodies. *F*, HEK-293T cells were cotransfected with plasmids encoding Myc-MGF505-9R, FLAG-RNF125, HA-Ub, and either V5-RNF125-WT or V5-RNF125-LD (LD: ligase dead) for 24 h, followed by a 4-h treatment with MG132. Subsequently, co-IP and immunoblotting were performed using the indicated antibodies. Co-IP, coimmunoprecipitation; HEK-293T, human embryonic kidney 293T cell line; RNF125, RING finger protein 125; Ub, ubiquitin.

To further investigate the specific mechanism by which pMGF505-9R antagonizes RNF125-mediated K48 ubiquitination of its substrates, we examined the influence of pMGF505-9R on the protein levels of RNF125 by cotransfecting varying doses of pMGF505-9R with RNF125 and detecting the expression of RNF125. The results showed that pMGF505-9R could inhibit the expression of RNF125 in a dose-dependent manner (Fig. 6, *A* and *B*). However, the overexpression of pMGF505-9R did not affect the mRNA levels of RNF125 (Fig. 6*C*). Previous studies have reported that the expression of RNF125 can be regulated by its autoubiquitination, subsequently leading to its degradation (26). This led us to speculate that pMGF505-9R may influence the ubiquitination of RNF125. As shown in Figure 6*D*, pMGF505-9R could increase the ubiquitination of RNF125. To demonstrate whether pMGF505-9R promotes the ubiquitination of RNF125 by leveraging RNF125's intrinsic E3 ubiquitin ligase activity, we generated an enzymatic activity–deficient mutant of RNF125 (27) and employed co-IP assays. The results revealed that loss of RNF125's enzymatic activity abolished the enhancement of RNF125 ubiquitination levels by pMGF505-9R (Fig. 6, *E* and *F*).

### ASFVΔMGF505-9R infection induced a weakened IFN response

**Figure 7:**
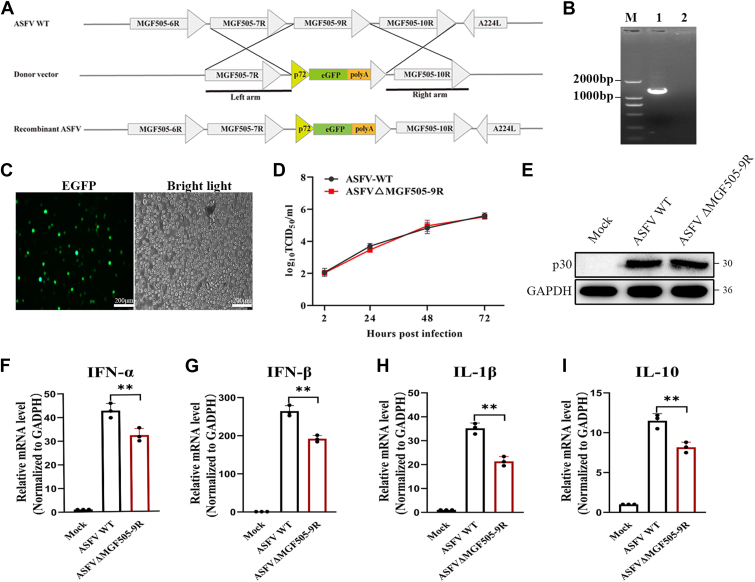
**ASFVΔMGF505-9R infection induced a lower IFN response.***A*, the ASFVΔMGF505-9R strain was generated *via* homologous recombination, as described in the Experimental procedures section. *B*, the MGF505-9R fragment was amplified from both the recombinant ASFV and its parental ASFV using specific primers. The PCR products were then analyzed by electrophoresis. *C*, the growth status of ASFVΔMGF505-9R in PAMs is depicted, with GFP indicating the presence of the virus. *D*, the replication characteristics of ASFVΔMGF505-9R and its parental virus were compared. PAMs were infected with either ASFVΔMGF505-9R or its parental virus (MOI = 0.1), and viral titers were measured at different time points. The TCID_50_ method was used to determine the viral titer. *E*, PAMs were infected with either ASFV or ASFVΔMGF505-9R (MOI = 1). At 24 hpi, the protein level of the viral protein p30 was detected by Western blotting. *F*–*I*, PAMs were infected with either ASFV or ASFVΔMGF505-9R (MOI = 1) and RNA was extracted from the cells at 24 hpi, the levels of IFN-α, IFN-β, IL-1β, and IL-10 mRNA were measured using qPCR. Scale bar represents 200 μm.

To verify whether pMGF505-9R promotes the production of IFN and inflammatory cytokines during viral infection, we constructed a recombinant ASFV lacking MGF505-9R by using homologous recombination (Fig. 7*A*). ASFVΔMGF505-9R was obtained without contamination from its parental ASFV by several rounds of purification and confirmed by amplifying the MGF505-9R gene coding sequence (Fig. 7*B*). Following the infection of PAMs with the ASFVΔMGF505-9R for 16 h, green fluorescence was observed (Fig. 7*C*). The replication characteristics of ASFVΔMGF505-9R were also evaluated in PAMs. The results showed no significant difference in viral titers between the gene-deleted strain and the parental strain (Fig. 7*D*). Subsequently, we examined the effect of the MGF505-9R gene deletion on the activation of innate immune signaling pathways during ASFV infection. Meanwhile, the protein level of the viral protein p30 was detected by Western blotting to verify the consistency of infection levels (Fig. 7*E*). It was found that, compared with the parental strain, the deletion of the MGF505-9R gene resulted in lower levels of mRNA expression for IFN-α, IFN-β, IL-1β, and IL-10 at 24 hpi (Fig. 7, *F*–*I*). These results indicated that ASFVΔMGF505-9R infection could elicit a weakened IFN response.

## Discussion

Numerous studies have demonstrated that ASFV encodes multiple proteins capable of suppressing innate immune signaling pathways, including the production of type I IFN and IFN signaling pathways (28, 29). Nevertheless, recent transcriptomic and proteomic analyses suggest that ASFV infection can trigger inflammation and cytokine storms, indicating a complex interplay between the virus and the host immune system (30, 31). Among other viruses, the M2 protein of the influenza virus positively regulates the host's innate immune response (32). It is still unclear whether the proteins encoded by ASFV could upregulate the innate immune signaling pathways. Therefore, we screened 160 proteins of ASFV and found that six proteins can positively regulate the promoter activity of IFN-β, with pMGF505-9R exhibiting the most pronounced effect. Further investigation revealed that pMGF505-9R has the capacity to upregulate the protein level of RIG-I, MDA5, and MAVS by modulating their K48-linked ubiquitination.

The ubiquitination of RIG-I, MDA5, and MAVS is regulated by multiple E3 ubiquitin ligases and deubiquitinating enzymes, which influence their activity and stability. For instance, the E3 ubiquitin ligase TRIM31 can directly catalyze the polyubiquitination of specific sites on MAVS, promoting its oligomerization and activating RLR signaling. Conversely, TRIM25 facilitates the degradation of MAVS by ubiquitinating MAVS at lysine residues 7 and 10, thereby inhibiting RLR signaling (33). Considering that pMGF505-9R has the ability to decrease the K48 ubiquitination level of RIG-I, MDA5, and MAVS, we hypothesized that pMGF505-9R may be able to antagonize the K48 ubiquitination of these proteins mediated by specific E3 ubiquitin ligases. After screening, we identified E3 ubiquitin ligase RNF125, which interacted with pMGF505-9R.

**Figure 8:**
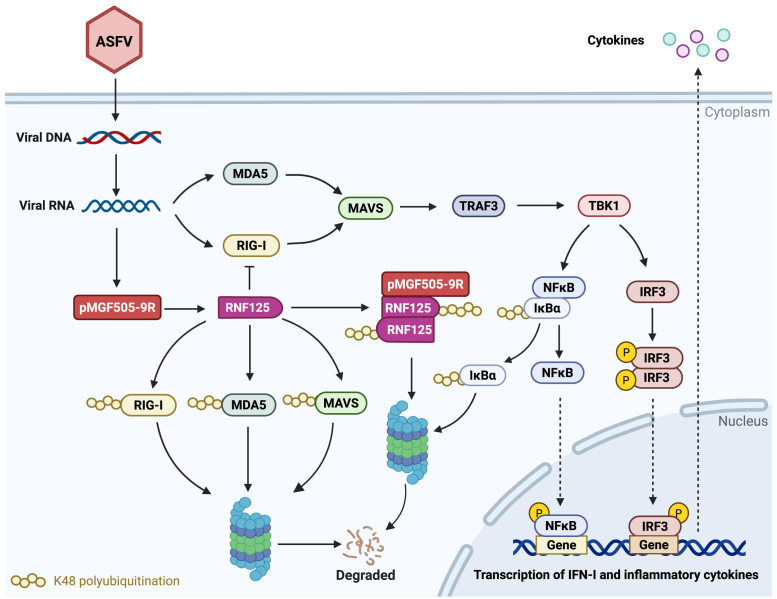
**Schematic overview of the mechanism model of pMGF505-9R regulates RIG-I-like receptor signaling pathway.** During viral infection, viral RNA is typically recognized by RIG-I, which subsequently activates MAVS and triggers a series of signaling reactions culminating in the production of type I interferon. To prevent an excessive response from this pathway, the negative regulatory factor RNF125 mediates K48 ubiquitination degradation of RIG-I, MDA5, and MAVS, thereby maintaining cellular homeostasis. However, ASFV pMGF505-9R can disrupt this process by promoting the autoubiquitination and subsequent degradation of RNF125, leading to increased stability of RIG-I, MDA5, and MAVS, ultimately enhancing the RLR innate immune signaling pathway. This schematic diagram was created using BioRender.com. ASFV, African swine fever virus; MAVS, mitochondrial antiviral signaling; MDA5, melanoma differentiation–associated protein 5; RIG-I, retinoic acid–inducible gene I; RLR, RIG-I-like receptor; RNF125, RING finger protein 125.

The RNF125 protein is an E3 ubiquitin ligase belonging to the RING finger protein family, which plays a crucial regulatory role in cellular processes. Research has demonstrated that RNF125 functions as a negative regulator of the RLR signaling pathway. RNF125 inhibits RLR-mediated signaling by interacting with key molecules in the RLR signaling cascade, thereby negatively regulating the antiviral response. When the RLR signaling pathway is activated, a substantial amount of cytokines is produced, which act on cells to upregulate the expression of the negative regulator RNF125 within the signaling pathway. Then, RNF125 induces the K48 ubiquitination and subsequent degradation of RIG-I, MDA5, and MAVS, thus maintaining intracellular homeostasis (34). In this study, we found that pMGF505-9R can decrease the protein levels of RNF125. It has been reported that RNF125 is capable of undergoing autoubiquitination, which leads to its degradation. Host factor G3BP1 plays a role in facilitating the autoubiquitination and degradation of RNF125 through interactions with RNF125. In our study, we found that the interaction between pMGF505-9R and RNF125 also promoted the autoubiquitination of RNF125 and subsequent degradation. These findings suggested that pMGF505-9R enhanced the RLR signaling pathway by promoting the ubiquitination and degradation of RNF125, as shown in Figure 8.

pMGF505-9R is a member of the MGF505 multigene family. Recent studies have demonstrated that pMGF505-7R inhibits the cyclic GMP–AMP synthase–STING signaling pathway, thereby suppressing the production of type I IFNs (35). Amino acid sequence alignment analysis revealed that the similarity between the amino acid sequences of pMGF505-9R and pMGF505-7R exceeds 30%. However, in the current study, pMGF505-9R exhibited a distinct function from pMGF505-7R. Instead of inhibiting the production of IFN-β, pMGF505-9R promoted the production of IFN-β by inducing the ubiquitination and degradation of RNF125. This finding suggested that members of the same gene family may achieve diverse immunomodulatory functions by targeting the same host factors. Typically, upon infection by pathogens, the innate immune system is activated, leading to the secretion of cytokines by cells. These cytokines recruit and activate additional innate immune cells to migrate toward the infected cells, facilitating the elimination of the infection. However, this mechanism appears to be ineffective against ASFV, as the virus infects various innate immune cells and employs strategies to evade macrophage-mediated clearance (36). Consequently, this process may promote the dissemination of ASFV.

While the majority of previous studies have reported that ASFV infection induces only low levels of IFN-β and IL-1β at 24 h postinfection, our findings demonstrated that infection with the ASFV CN/JS-1/2024 strain triggered significantly elevated levels of IFN-β and IL-1β mRNA at the same time point. This discrepancy may reflect critical genomic differences between viral strains, particularly in immune-modulatory genes, such as MGF505 and A238L. Yet, it is still unclear whether the virus-encoded proteins play a certain role in the increased production of cytokines during the late stage of virus replication. Our research revealed that pMGF505-9R facilitates the ubiquitination and degradation of the negative regulator RNF125 within the IFN-β production pathway, thereby leading to the increased production of cytokines. As a result, the infected cells could be more susceptible to recognition and phagocytosis by activated macrophages, which may ultimately facilitate viral infection.

In summary, we identified that ASFV pMGF505-9R positively regulates the RLR signaling pathway. As we know, this study reports for the first time that ASFV possesses a novel mechanism that positively regulates the host innate immune signaling pathway.

## Experimental procedures

### Cells and viruses

Human embryonic kidney 293T (HEK-293T) cells were utilized for co-IP experiments owing to their high transfection efficiency and robust protein expression. ST cells (porcine testis cell line) were employed for signaling analyses given their well-characterized IFN response pathways. Both cell lines were cultured in Dulbecco's modified Eagle's medium (Gibco; catalog no.: 11965-092) supplemented with 10% fetal bovine serum (Lonsera; catalog no.: S711-001) at 37 °C in a 5% CO_2_ incubator. PAMs were selected for viral infection studies because of their physiological relevance as primary targets of ASFV *in vivo*. They were cultured in RPMI1640 medium (Gibco; catalog no.: C22400500BT) containing 15% inactivated fetal bovine serum and maintained at 37 °C in a 5% CO_2_ incubator. The recombinant Sendai viruses encoding enhanced GFP and VSV encoding eGFP were constructed and stored in our laboratory. The ASFV strain, ASFV CN/JS-1/2024 (GenBank accession number: PV541693) was isolated from field samples from China. All experiments with live ASF viruses were conducted within the biosafety level 3 (P3) facilities at the Yangzhou University of the Chinese, approved by the Ministry of Agriculture and Rural Affairs.

### Construction of the recombinant ASFV using the homologous recombination method

The recombinant virus was created through homologous recombination, as previously described (37). Briefly, PAMs were transfected with the donor vector that contained the recombinant cassette using Lipofectamine 3000 transfection reagent (Thermo Fisher Scientific) for 4 h. The cells were then infected with ASFV CN/JS-1/2024. After 48 h, cells expressing GFP were selected and then inoculated onto a culture plate with healthy cells. At 48 hpi, the supernatant was collected and used to reinfect healthy cells. This purification step was repeated several times using the same method until all the infected cells expressed green fluorescence, and the supernatant obtained was the purified virus. The purified virus was then identified using PCR with specific primers targeting MGF505-9R. The primer sequences were as follows: MGF505-9R-F: 5′-ATGTTCTCCCTACAGGATCTCTGTC-3′, and MGF505-9R-R: 5′-TACTTAAATGTAATAAGATCTATATATTTGATACATTCATCTGCG-3′. The viral titers of the parental strain ASFV CN/JS-1/2024 and the recombinant virus ASFV-ΔMGF505-9R were determined by measuring TCID_50_.

### Plasmids, reagents, and antibodies

The coding sequences of 160 ASFV genes were amplified from viral genomic DNA and cloned into the pCAGGS expression vector with an N-terminal 3× FLAG tag. Commercial reagents were obtained as follows: DDDDK-Tag (AE005), HA-Tag (AE008), Myc-Tag (AE010), RNF125 (A15166), RIG-I (A0550), and GAPDH (A19056) were purchased from ABclonal Technology; phospho-P65 (82335-1-RR), P65 (10745-1-AP), phospho-IRF3 (29528-1-AP), MDA5 (21775-1-AP), MAVS (14341-1-AP), TBK1 (28397-1-AP), and IRF3 (66670-1-Ig) were purchased from Proteintech. All antibodies were validated for crossreactivity with porcine cellular components and demonstrated applicability for detecting porcine-derived proteins. Poly(I:C) (CAS 24939-03-5) was sourced from MedChemExpress, whereas the monoclonal antibody against ASFV p30 protein was developed and maintained in-house. Plasmid transfection was performed using polyethyleneimine (Proteintech; B600070) according to the manufacturer's standardized protocol.

### Dual-luciferase reporter activity assays

HEK-293T cells cultured in 48-well plates were transfected with 0.1 mg/well of a luciferase expression plasmid, along with 0.01 mg/well of pRL-TK and plasmids expressing viral proteins. At 24 h post-transfection, the cells were transfected with 2 μg/ml of poly(I:C) or mock-treated for an additional 12 h. Subsequently, the cells were lysed, and the activities of firefly and Renilla luciferases in the lysates were analyzed using the Dual Luciferase Assay Kit (TransGen Biotech; catalog no.: FR201-01-V2) according to the manufacturer's protocol.

### Immunofluorescence staining

ST cells were plated on coverslips in 24-well plates and subsequently infected with the indicated viruses or transfected with the specified plasmids. At 24 h post-transfection, the cells were fixed with 4% paraformaldehyde for 15 min, followed by permeabilization and blocking with 0.1% Triton X-100 (Sigma–Aldrich; catalog no.: X100) and 1% bovine serum albumin in PBS for 30 min at room temperature. The cells were then incubated with the corresponding primary antibodies at 4°C overnight. Afterward, the cells were treated with 488 nm and 594 nm conjugated goat anti-mouse or anti-rabbit secondary antibodies for 1 h. Nuclei were stained with 4’-6-diamidino-2-phenylindole (Invitrogen; catalog no.: 00-4959-52) for 10 min. After each step, all cells were washed with PBS three times before being imaged using an inverted fluorescence microscope and confocal microscopy.

### RNA isolation and PCR analysis

To determine the effect of the pMGF505-9R protein on the expression levels of IFN-β and IL-1β, ST cells seeded in 24-well plates were transfected with either the empty vector or the FLAG-tagged MGF505-9R construct. After 24 h post-transfection, the cells were either mock-treated or transfected with poly(I:C) for an additional 12 h. To assess the influence of the pMGF505-9R protein on MAVS or RNF125, plasmids encoding MGF505-9R were transfected into the cells for 24 h. Total RNA was extracted using TRIzol (Invitrogen; catalog no.: 15596018) according to the manufacturer’s instructions, and 1 μg of RNA was reverse transcribed. Gene expression was quantified using SYBR Green–based real-time RT–PCR. Each gene expression level was normalized to that of GAPDH. The primers used in this study are presented in Table 1.

**Table 1:** The sequences of the primers used for PCR

Primer	Sequence (5′-3′)	Description
pIFN-β-F	GCTAACAAGTGCATCCTCCAAA	Porcine IFN-β gene
pIFN-β-R	AGCACATCATAGCTCATGGAAAGA	
pTNF-α-F	ATGAGCACTGAGAGCATGATCCG	Porcine TNF-α gene
pTNF-α-R	CGAAGTGCAGTAGGCAGAAGAG	
pIFN-α-F	TGCTCTCTGGGCTGTGACC	Porcine IFN-α gene
pIFN-α-R	CCCAAAAGCCTCATGAGGGG	
pIL-1β-F	ACCCAAAACCTGGACCTTGG	Porcine IL-1β gene
pIL-1β-R	CATCACAGAAGGCCTGGGAG	
pIL-6-F	CTCATTAAGTACATCCTCGG	Porcine IL-6 gene
pIL-6-R	GTCTCCTGATTGAACCCAGA	
pGAPDH-F	ACATGGCCTCCAAGGAGTAAGA	Porcine GAPDH gene
pGAPDH-R	GATCGAGTTGGGGCTGTGACT	
pIL-10-F	GCTCTATTGCCTGATCTTCCTGGC	Porcine IL-10 gene
pIL-10-R	AACTCTTCACTGGGCCGAAGG	
pMAVS-F	TGCCTCACAGCAAGTGACCA	Porcine MAVS gene
pMAVS-R	GGAATTCAAACAGCCCCCCA	
pRIG-I-F	ATGACAGCAGAGCAGCGG	Porcine RIG-I gene
pRIG-I-R	CAGCCTTCCTCCTGGAGCTC	
pMDA5-F	ATGTCGTCGGATGGGTATTCC	Porcine MDA5 gene
pMDA5-R	CTGGGAACATAAACGCAGCTGAAC	

### Coimmunoprecipitation

HEK-293T cells or ST cells were cultured in 10-cm dishes, and the monolayer cells were transfected with the indicated plasmids. After collection and lysis, the lysates were immunoprecipitated using the specified antibody as described previously. For Western blotting, the target proteins were resolved by 10% SDS-PAGE and then transferred onto a polyvinylidene fluoride membrane (Thermo Scientific). The membrane was blocked with 5% skim milk in Tris-buffered saline with Tween-20 at room temperature for 2 h, followed by incubation with the appropriate primary antibody at 4 °C overnight. Subsequently, the corresponding secondary antibody was incubated at room temperature for 2 h. Antibody–antigen complexes were visualized using chemiluminescence detection reagents (Thermo Scientific).

### Statistical analysis

Statistical tests were performed using GraphPad Prism, version 9.0 (GraphPad Software, Inc). The significance of differences between samples was assessed using an unpaired two-tailed Student’s *t* test. Variance was estimated indirectly by calculating the SD, and this is represented by error bars. Statistical significance was denoted as follows: ∗*p* < 0.05; ∗∗*p* < 0.01; and ∗∗∗*p* < 0.001.

## Data availability

All data are available from the corresponding author upon reasonable request.

## Conflict of interest

The authors declare that they have no conflicts of interest with the contents of this article.
